# Papillary Thyroid Carcinoma and Its Hormonal Effects in Women Following Surgical Removal

**DOI:** 10.7759/cureus.91784

**Published:** 2025-09-07

**Authors:** Sophia Salazar, Eric G Yang, Vindhya N Reddy, Savithri-Chandana Veluri

**Affiliations:** 1 Biology, Justin-Siena High School, Napa, USA; 2 Biology, Brown University, Providence, USA; 3 Medicine, Apollo Institute of Medical Sciences and Research, Hyderabad, IND; 4 Peri-Operative Medicine, Orlando Veterans Affairs Medical Center, Orlando, USA; 5 Women's Health, Orlando Veterans Affairs Medical Center, Orlando, USA

**Keywords:** bibliometric analysis, cancer research, papillary thyroid cancer, thyroid cancer, thyroidectomy, thyroid hormone therapy

## Abstract

While typically viewed as indolent, papillary thyroid carcinoma (PTC) is a clinically significant health condition, especially among women, as the primary treatment for PTC, thyroidectomy, and disproportionately affects them. Thyroidectomy disrupts endocrine function, often necessitating a lifetime of hormone replacement therapy, yet postoperative hormonal outcomes remain understudied. PTC's global research trends, post-treatment effects, and treatment methods are the primary focus of this bibliometric analysis, as the growing awareness of PTC and medical drug toxicity continues to stimulate medical research.

Bibliometric methods were used to evaluate trends in PTC-related publications from 2016 to 2025, acquiring data from the Web of Science database. The database searched for the following keywords: “papillary thyroid carcinoma”, “thyroidectomy”, “hormone replacement therapy”, and “women’s health.” Data were downloaded and analyzed using VOSviewer (Leiden University, Leiden, The Netherlands), a bibliometric network creation and visualization software. The qualitative analysis included analysis of co-occurrence of specific keywords in and between publications, common co-authorships, analysis of national collaboration networks, and thematic mapping.

Results indicated that the literature primarily focuses on PTC and surgical treatments while neglecting endocrine outcomes, hormonal challenges, and postoperative quality of life. The keywords "surgery" and "management" are widespread, whereas "levothyroxine" and "replacement therapy" are rarely used. Despite the thyroid gland’s central position in endocrine control, few studies connect treatment of PTC with any systemic hormonal effects. Moreover, international collaboration is highest between France, the People’s Republic of China, and the United States, with the same nations also topping PTC publication figures. A growing interest is shown by the increasing number of publications.

Throughout, the bibliometric analysis finds both a notable scarcity of literature on hormonal outcomes in female patients with PTC who have been treated with thyroidectomy and an abundance of surgical literature on common treatment methods for PTC. Unlike existing bibliometric studies, this paper centers on gender-specific consequences and underrepresented endocrine outcomes, offering new insights into long-neglected dimensions of PTC care.

## Introduction and background

The thyroid’s primary function within the body is to create and secrete two distinct types of hormones: thyroxine (T4) and triiodothyronine (T3). T4 is essential for regulating metabolism, growth, and development, and also serves as a major precursor to T3, a more active thyroid hormone with effects in nearly all bodily systems, primarily acting on metabolism and how the body uses energy [1]. T3 is essential for growth, brain development, maintaining a steady heart rate, and ensuring optimal muscle function [2]. Additionally, the thyroid secretes other hormones, including calcitonin, which regulates blood calcium levels and plays an influential role in maintaining bone health [3].

The thyroid produces thyroid hormones that are crucial for regulating metabolic processes in women, such as the menstrual cycle, fertility, and pregnancy. If thyroid hormone levels become irregular, women may experience irregular menstruation, difficulty conceiving, or complications during pregnancy, such as miscarriage or preterm labor. Additionally, women are more predisposed to developing thyroid cancers than men, and given the role thyroid hormones play in female reproductive health, it is extremely important to continue the research in this field [4].

Papillary thyroid carcinoma (PTC) often originates in one lobe of the thyroid gland, in the follicular cells, but often has the potential to metastasize to nearby lymph nodes [5]. While the precise etiology remains unknown, it is generally acknowledged that PTC accounts for approximately 85% of all thyroid cancers and is classified as an endocrine malignancy [6]. When comparing PTC tissue to wild-type samples, researchers noted that PTC differed from some cancers in that it involved numerous complex changes in how genetic information is processed, rather than just isolated mutations. This may help explain why some biomarkers do not work as effective diagnostic tools for some patients and further cites the need for more personalized approaches in diagnosis and treatment [7]. 

Although PTC is regarded as less aggressive than other thyroid cancers, it still retains the potential to spread to other lymph nodes within the cervical region. It may also spread to the bone, resulting in pain and long-term complications that may further affect patients’ quality of life, even after treatment [8]. Common manifestations associated with PTC include swollen lymph nodes, masses in the neck, dysphagia, dysphonia, and cervical pain [9]. PTC's pathophysiology reduces the overall efficiency of the thyroid gland, causing many of the associated symptoms [10]. The tumor grows inside the thyroid, thus interfering with the gland’s ability to produce thyroid hormones. Treatments include thyroidectomy (surgical removal), radioiodine therapy, and thyroid hormone replacement. 

Thyroidectomy is often preferred because it can remove the tumor entirely [11]. However, while the procedure removes all cancerous tissue, it also eliminates the gland’s hormonal function, causing the need for extensive postoperative management [12]. As women are more genetically predisposed to developing thyroid-related complications, thyroidectomy consequently affects them disproportionately in comparison to male patients. It is often necessary for many patients to take lifelong thyroid hormone supplementation to compensate for the surgery.

This study uses bibliometric methods to explore the research surrounding PTC. Furthermore, the analysis will help to identify areas that may have been inadequately addressed in order to improve the understanding of the global research landscape regarding PTC treatment, the trends in medical literature, and postoperative side effects of treatment.

## Review

Methods 

Bibliometric analysis is a method used to analyze large numbers of publications to identify trends and gain insights. The main goal of the bibliometric analysis is to offer a deeper understanding of how a research field has developed and how it is structured. Using citation analysis, bibliometric analysis can help identify emerging research themes, collaboration networks, and gaps in the literature that warrant further exploration [13]. Bibliometrics offer a way to systematically evaluate large volumes of scholarly literature to comprehend the evolution and existing structure of the research field. This analysis also identifies emerging research themes, collaboration networks, and areas requiring additional research.

Search Strategy

Data were obtained from the Web of Science (WOS) Core Collection database, using the following search: “papillary thyroid cancer OR papillary thyroid carcinoma (Topic) AND treatment (Topic) and 2025 or 2024 or 2023 or 2022 or 2021 or 2020 or 2019 or 2018 or 2017 or 2016 (Publication Years).” The search was limited to articles written in English published between 2016 and 2025, with the motive of keeping the focus on recent developments in the area. Articles and publications were further organized by their relevance to either the medicinal, hormonal, or treatment aspects of PTC.

Data Visualization

The data were visualized using VOSviewer (Leiden University, Leiden, The Netherlands), which was used to create keyword co-occurrence maps, citation analysis, and collaboration visualizations between countries and institutions. The WOS data were put through VOSviewer’s clustering algorithm, which uses modularity optimization to generate figures that group closely related items. A minimum threshold for keyword occurrences was set at 5, based on relevance and map readability, to exclude infrequent topics and distinguish prominent research themes. The visualization allowed the identification of core topics, frequent terms, and under-explored areas in the literature. In so doing, the study aimed to explore not only the most popular areas of study in PTC but also overlooked topics.

Results

**Figure 1:**
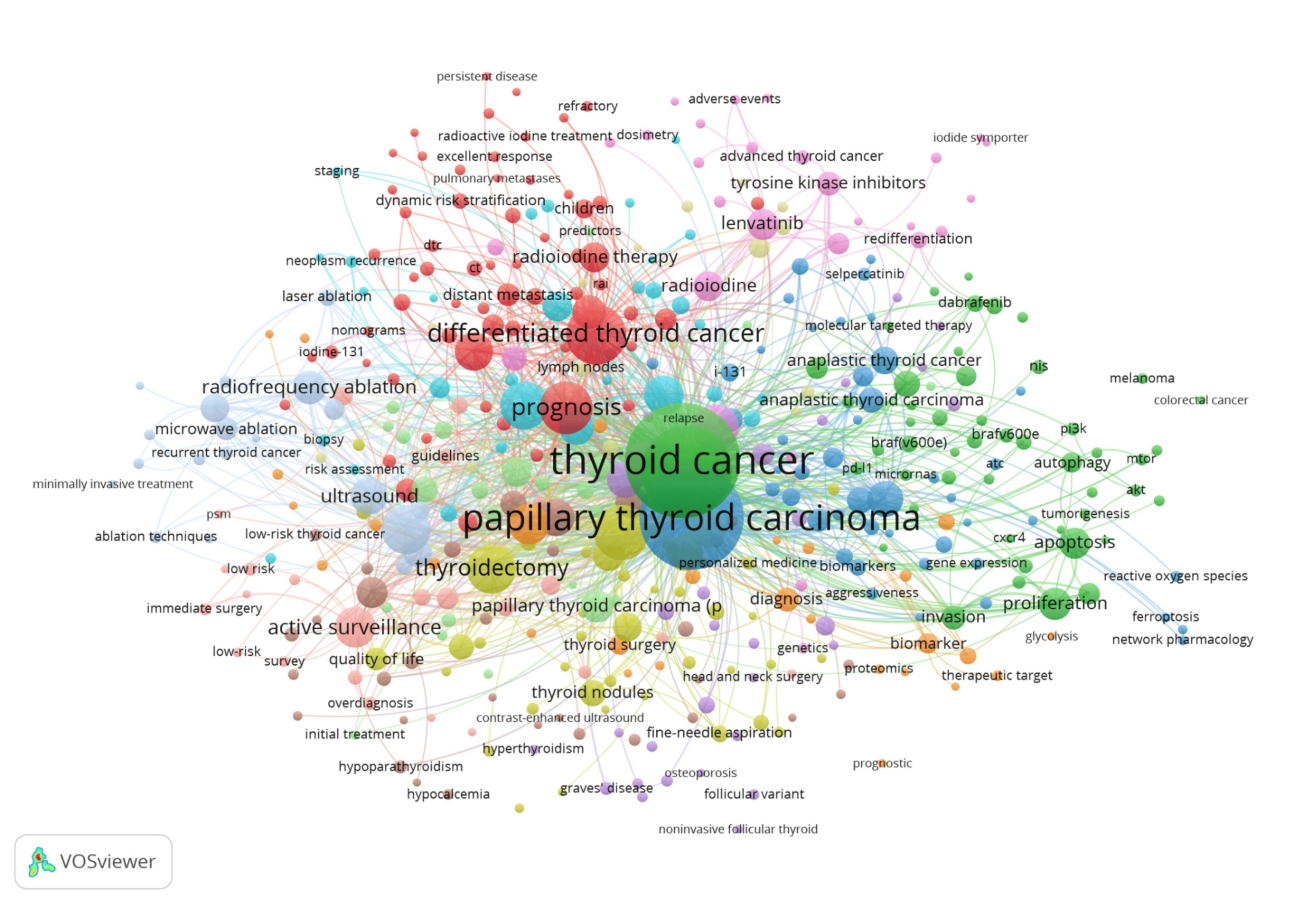
Co-occurrence of author keywords

Figure 1 depicts the co-occurrence of keywords that the authors chose to describe their articles with. The analysis displays how often author-supplied keywords appear together in a collection of publications. It shows “thyroid cancer” in the center and “papillary thyroid cancer” as the main variant of thyroid cancer. The size of the circle represents the frequency with which a term appeared in the text, with “thyroid cancer” as the most frequent, which is expected, given the initial search parameters. “Papillary thyroid cancer” is a close second, likely since it accounts for the majority of thyroid cancers. It shows that topics such as quality of life, radioiodine therapy, and adverse events are less frequently discussed. Conversely, topics such as thyroidectomy are heavily emphasized. However, analysis reveals that the keyword “prognosis” is relatively common, indicating a growing interest in patient outcomes. “Radioiodine therapy” and “adverse effects”, although topics of much importance concerning patient care, are significantly less emphasized. 

**Figure 2:**
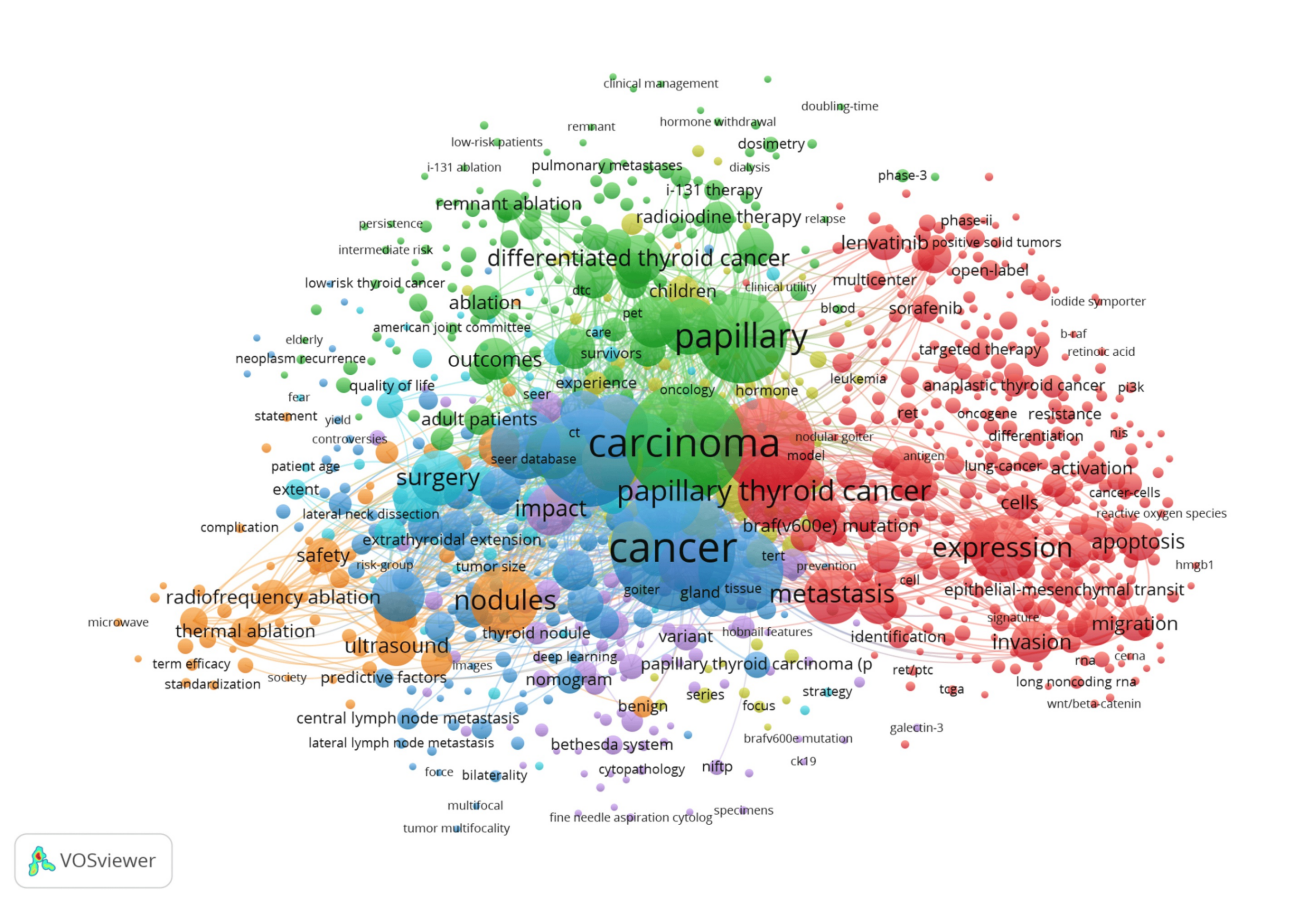
Co-occurrence of all keywords

Figure 2 illustrates the relationship between keywords found in a collection of documents, based on how frequently those keywords appear together within each publication. “Cancer”, “papillary”, and “carcinoma” are the most frequently used words in publications. Analysis also reveals another instance of surgery being a primary focus of PTC publications, as observed by the size of the “surgery” node. The number of publications for keywords such as “hormone withdrawal” and “hormone” is relatively low; however, their presence could be indicative of increasing research in the area. Notably, there is no connection between hormone withdrawal and any of the three most prominent keywords. 

**Figure 3:**
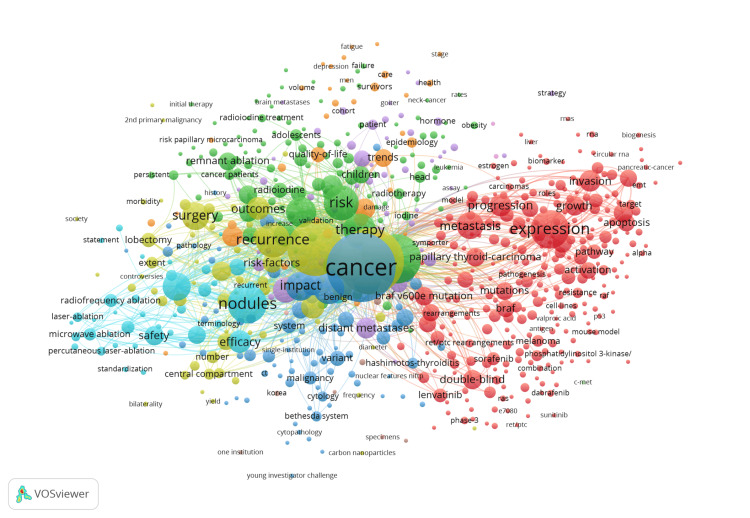
Co-occurrence of keywords

Figure 3 depicts the relationships between common keywords that occur over a set of publications; it represents a map of research topics and their common connections within the WOS dataset. Figure 3 is a set of automatically generated keywords that are derived from the titles of cited references within a document. Topics such as therapy, surgery, and impact are heavily emphasized due to their traditional connection to cancer research. Other topics, such as hormones, are scarcely mentioned in the acquired literature, and analysis reveals that these topics rarely have any notable connection to any major topic concerning papillary thyroid carcinoma.

**Figure 4:**
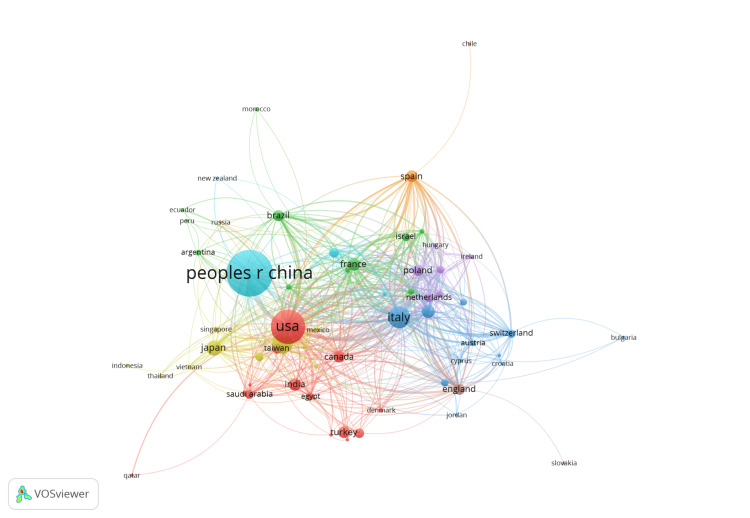
Co-authorship by country

Figure 4 highlights the countries that collaborate and publish most frequently, revealing patterns of international research cooperation. The size of the circle represents the prevalence of research within a country. China has the most publications, with the United States and Italy picking up the next two spots, respectively. This could be due to the prominence these countries have in medical research. Figure 4 also shows a strong web of connections between all participating countries except for Slovakia, Bulgaria, Qatar, Chile, and Indonesia. This indicates a strong international network for collaboration on PTC, showing global interest and efforts to advance research on the disease.

**Figure 5:**
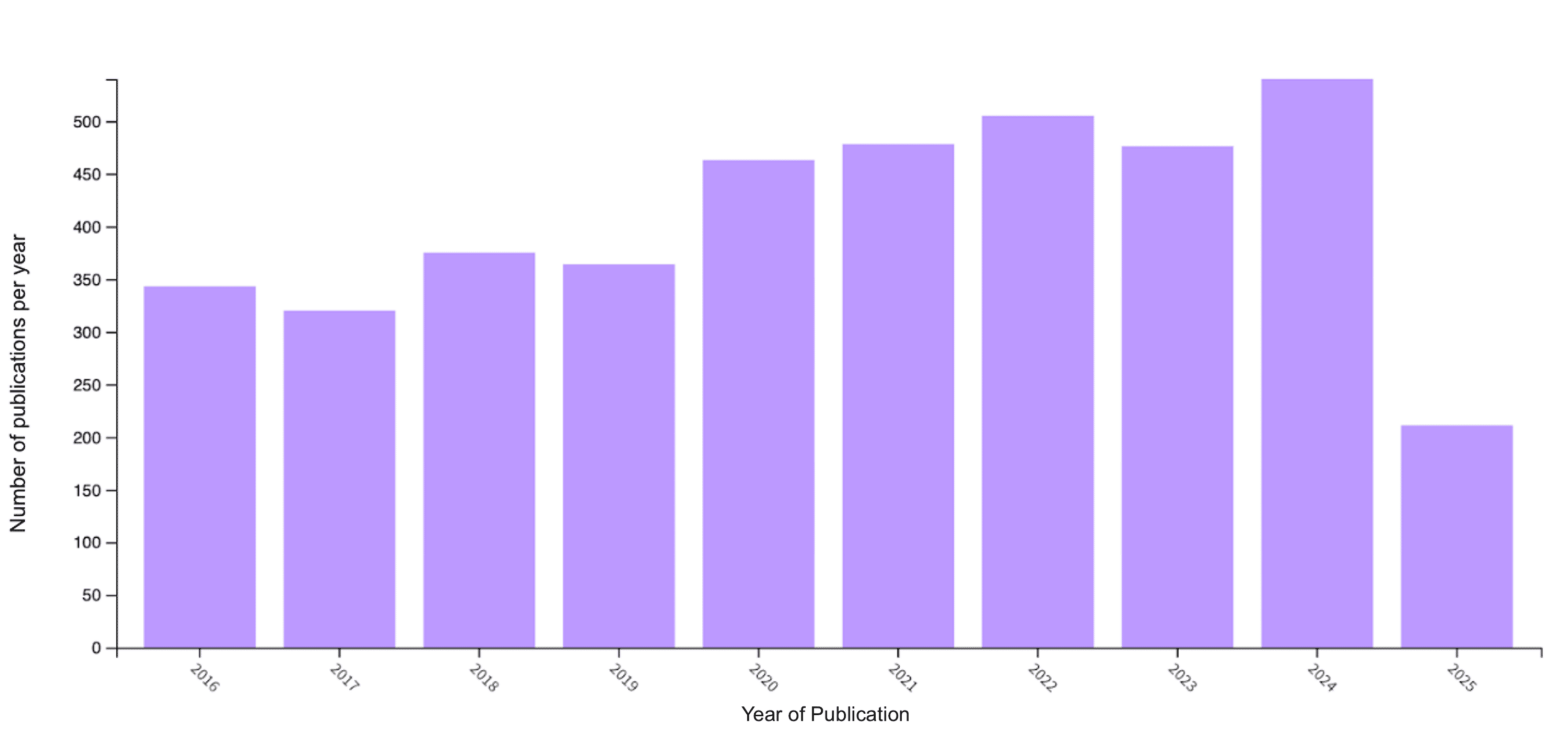
Number of papillary thyroid carcinoma publications per year (2016-2025)

Figure 5 demonstrates a relatively constant number of publications between 2016 and 2025, with 2024 having the highest number of publications on the topic. A limitation of Figure 5 is its restricted time range, which may obscure broader patterns. The trends in Figure 5 indicate a stagnating growth of interest in PTC. The year 2025 has fewer publications because this study was conducted in the middle of the year.

Discussion

In the visual representation of keyword occurrences, “carcinoma” emerges as the primary area of research, while “papillary” is slightly less studied. Surgery is the treatment with the most number of publications. This is in line with thyroidectomy being the standard of care for PTC. 

Adverse effects of thyroidectomy were not prominent in this analysis. This highlights an important issue in the research landscape, since the consequences of thyroidectomy can be as impactful as the disease itself. Furthermore, words relating to endocrine supplements such as "levothyroxine" and "replacement therapy" do not appear in many studies, despite their clinical relevance in female patients with PTC [14]. This may be due to the lack of incorporating keywords "quality of life" or "adverse effects of thyroidectomy" in the search. 

This finding also aligns with the concerns about gender equity in healthcare, as thyroid cancer disproportionately affects women, yet their experiences are rarely documented in the reviewed literature [15,16]. Current literature avoids controversial issues such as overtreatment or delayed intervention for low-risk patients, despite their presence in clinical debates [17]. Similarly, the bibliometric study shows a limited number of studies concerning “low-risk patients”. Initial treatment is significantly less studied. 

In examining international collaboration, it is observed that the People's Republic of China and the United States frequently collaborate with other nations. An analysis of country and keyword patterns suggests that these two countries focus more on surgical approaches, while European countries show slightly more engagement with hormone-related topics. The dense connections between European and Asian countries suggest consistent global cooperation, yet they appear only to reinforce dominant clinical perspectives rather than expanding the focus to include less-studied topics. 

In the annual publication chart, the visualization indicates a slight increase in interest, although it is not statistically significant. Notably, 2024, the most recent full year, has the highest number of publications observed in the past decade, possibly indicating a growing focus on PTC and the potential for further exploration of its less-studied aspects.

There are several alternatives to total thyroidectomy that may offer patients less invasive options with fewer long-term consequences. For instance, a lobectomy, which removes only part of the thyroid, can help some patients retain enough thyroid function to avoid lifelong hormone replacement therapy [18]. Active surveillance, especially common in parts of Latin America, is gaining traction as a safe option for patients with small, slow-growing tumors that may never progress [19]. In addition, personalized treatment plans, guided by genetic and molecular profiling, are making it possible to tailor therapies to individual patient needs, helping to minimize unnecessary procedures while targeting the cancer more precisely [20]. Despite their potential, these alternatives remain under-researched in the current research landscape.

Limitations 

One limitation is the exclusive use of VOSviewer for figure generation, which may result in discrepancies in the results due to its limitations when handling larger networks. It is also important to note that VOSviewer notes solely publication volume and does not account for local barriers to publication, such as funding limitations, language access, and institutional support. 

A common limitation in bibliographic studies is selection bias, since the paper sources all its data from the WOS, which, while still having a large amount of data, could dilute the validity of the findings. Additionally, the time frame for the figures was restricted to the past 10 years, which obstructs a comprehensive understanding of the situation. The analysis in VOSviewer was conducted in English, thus creating a publication bias and limiting the number of papers that could be included in the study. Finally, significance testing or confidence intervals are not reported, which is common in bibliometric analyses but nonetheless a limitation. 

Future directions

Future research could include clinical, hormonal, and psychosocial dimensions to offer integrative, gender-sensitive care. Additionally, the study finds a direct relationship between cross-functional collaboration and publication of post-treatment studies, citing an essential overlap in the development of the field. Although PTC presents as a lower-risk cancer with a relatively low death rate and high success rate, the study also cites the overlooked need for increased research into less debilitating methods of treatment for PTC to treat postoperative effects in women.

## Conclusions

This bibliometric analysis discusses the current research landscape on PTC. The study reveals that academic literature is unbalanced, as surgical interventions dominate, while academic literature on endocrine, quality of life, and postoperative hormonal challenges remains scarce. It is demonstrated that "patient experience", "endocrine system disruption", and "hormone withdrawal" keywords are greatly underrepresented, presenting a dangerous disparity due to their critical importance in adequate patient outcomes. This is an immensely concerning issue for women, especially given their genetic predisposition for the disease. 

The results also show a high level of international activity, with the People's Republic of China, the United States, and Italy being the leading producers. Yet, the research’s emphasis continues to be disproportionately skewed toward surgical intervention instead of developing patient-oriented care. Despite a steady increase in publication volume throughout the last decade, some of the most significant issues in long-term management, especially those influencing women’s health and enforcing a status, continue to be understudied. 
